# Supplementary material for: Mechanisms governing the pioneering and redistribution capabilities of the non-classical pioneer PU.1
Source: Nat Commun. 2020 Jan 21;11:402. doi: 10.1038/s41467-019-13960-2 (PMC6972792; doi:10.1038/s41467-019-13960-2)
Supplement: Supplementary file 7 — Source data [file 41467_2019_13960_MOESM7_ESM.zip › Source_Data/FigureS2/FigureS2B_MotifScanOutput/KmeansCluster10/homerResults/motif23.similar.html]

motif23

## Information for motif23

C
A
T
G
A
G
T
C
C
G
A
T
A
C
T
G
A
G
T
C
T
G
A
C
T
G
C
A
  
Reverse Opposite:  

A
C
G
T
A
C
T
G
T
C
A
G
A
G
T
C
G
C
T
A
C
T
A
G
G
T
A
C
  

|  |  |
| --- | --- |
| p-value: | 1e-28 |
| log p-value: | -6.473e+01 |
| Information Content per bp: | 1.787 |
| Number of Target Sequences with motif | 1377.0 |
| Percentage of Target Sequences with motif | 37.10% |
| Number of Background Sequences with motif | 12508.5 |
| Percentage of Background Sequences with motif | 28.30% |
| Average Position of motif in Targets | 98.0 +/- 57.1bp |
| Average Position of motif in Background | 100.0 +/- 63.8bp |
| Strand Bias (log2 ratio + to - strand density) | -0.0 |
| Multiplicity (# of sites on avg that occur together) | 1.25 |
| Motif File: | file (matrix) reverse opposite |

### Similar de novo motifs found

|  |  |  |  |  |  |  |  |
| --- | --- | --- | --- | --- | --- | --- | --- |
| Rank | Match Score | Redundant Motif | P-value | log P-value | % of Targets | % of Background | Motif file |
| 1 | 0.925 | G C T A C A T G T G A C C G A T A C T G T A G C G T A C C G T A | 1e-25 | -58.319601 | 36.05% | 27.77% | motif file (matrix) |
| 2 | 0.883 | A C T G A C G T C A G T T A C G A C T G A T G C C G T A A C T G A G T C | 1e-17 | -40.561566 | 7.25% | 4.00% | motif file (matrix) |
